# Comparing the impacts of different exercise interventions on patients with type 2 diabetes mellitus: a literature review and meta-analysis

**DOI:** 10.3389/fendo.2025.1495131

**Published:** 2025-05-05

**Authors:** Shuangtao Xing, Yifan Zhang, Yanjiao Chen, Shijie Feng, Yiqing Zhang, Paulo Moreira

**Affiliations:** ^1^ Physical Education Institute of Henan Normal University, Henan, Xinxiang, China; ^2^ Department of Public Basic Education of Henan Vocational University of Science and Technology, Henan, Zhoukou, China; ^3^ Research Center for Social Work and Governance, College of Social Affairs, Henan Normal University, Henan, Xinxiang, China; ^4^ International Healthcare Management Research & Development Center (IHM-RDC), First Affiliated Hospital of the Shandong First Medical University and Shandong Provincial Qianfoshan Hospital, Jinan, Shandong, China; ^5^ Atlantica Instituto Universitario, Gestao em Saude, Oeiras, Portugal

**Keywords:** exercise interventions, type 2 diabetes mellitus, diabetes management, healthcare management, meta-analysis

## Abstract

**Objective:**

Exercise interventions are a recommended method of diabetes management through which patients can achieve blood glucose control, increase muscle volume, and improve insulin sensitivity, while also improving blood lipids, blood pressure, and cardiovascular health. A few studies on the effects of physical exercise on diabetic patients have been published in recent years. This article focuses on exploring evidence on which exercise interventions generate which effects in diabetic patients, namely, high-intensity interval training (HIIT), method training (MT), aerobic exercise training (AET), resistance training (RT), and combined training (CBT).

**Methods:**

Randomized controlled trials (RCTs) that focused on the effects of exercise interventions on blood glucose and blood lipids of patients with type 2 diabetes mellitus were reviewed. A network meta-analysis was performed to compare the effects of the five exercise interventions in diabetic patients, namely the impacts on glycosylated hemoglobin (HbA1c), fasting blood glucose (FBG), total cholesterol (TC), triglycerides (TG), high-density lipoprotein (HDL), and low-density lipoprotein (LDL). The study was strictly conducted following the PRISMA Protocol, and the Cochrane Risk of Bias Assessment Tool 2.0 was used to objectively evaluate the risk of bias in the implementation of the study.

**Results:**

This review included 25 RCTs in total, with 1,711 subjects. Meta-analysis suggests that, compared with conventional therapeutic treatment, exercise interventions can reduce blood glucose indexes, namely HbA1c, FBG, TC, TG, HDL, and LDL. RT and AET have been shown to reduce TC; HIIT, MT, AET, and CBT have been shown to improve HDL; and HIIT, MT, AET, and CBT have been shown to improve HDL. The MT and RT exercise types can reduce LDL. Evidence also suggests that MT can lower HbA1c, TG, and LDL levels, and RT lowers cholesterol levels. HIIT exercise appears to improve FBG and HDL levels.

**Conclusion:**

The five types of exercise generate different effects on the key clinical dimensions of diabetes. MT seems to be the optimal choice to improve HbA1c, TG levels, and LDL, while HIIT improves FBG and HDL levels, whereas RT exercise appears to be the optimal exercise to lower cholesterol levels.

## 1 Introduction

Type 2 diabetes mellitus (T2DM) is a metabolic disease characterized by chronic hyperglycemia. The pathogenesis of T2DM is insulin resistance and lack of insulin secretion, but genetic and environmental factors can also increase the risk of developing it (1). It is a complex disease involving almost all organs and systems of the body and is closely related to obesity and metabolic syndrome, which is one of the most serious chronic diseases threatening human health (2). According to the data of the 7th National Census of China National Bureau of Statistics (3), approximately 30% of elderly people suffer from diabetes, with T2DM accounting for over 95%, and more than half of patients with diabetes have low levels of blood glucose control and are commonly diagnosed with diabetes at the same time as diabetic complications or ischemic cardiovascular disease. Furthermore, 30%–40% of elderly patients have a combination of glucose metabolism disorders, hypertension, abdominal obesity, and hypertriglyceridemia due to mutations in the lipoprotein lipase gene (4), which refers to a condition in which there is an abnormally high level of triglycerides (TG) in the blood. The WHO predicts that by 2030 diabetes will be ranked as the seventh leading cause of death (5, 6).

There are two main kinds of treatment approaches for diabetes: therapeutic options and non-therapeutic options. The therapeutic approach lowers glucose levels with the disadvantage of side effects and adverse reactions. Non-pharmaceutical interventions, with their simplicity, potential efficacy, and good levels of patient safety, are gradually garnering attention in the healthcare community. In integrated diabetes management (7), blood glucose control is recognized as a primary strategy for diabetes management, and blood lipid control is an additional important measure to prevent and treat cardiovascular disease in patients with T2DM. A number of studies on the effect of physical exercise on diabetic patients have been published internationally in recent years. Among the consistent conclusions of published research is that a lack of exercise is one of the risk factors for diabetes. Additionally, exercise interventions are a recommended rudimentary method for diabetes management (8), by which diabetic patients can achieve blood glucose control by directly consuming some energy through exercise. Physical exercise can also increase muscle volume, regulate the function of the cerebral cortex, influence the thalamic-pituitary-target gland system, and improve the function of the patient’s islet beta cells, promoting increased insulin secretion (9). Furthermore, exercising promotes skeletal muscle glucose uptake, improves insulin sensitivity and insulin resistance, and elevates glucose transport. This series of reactions can effectively achieve the objective of stabilizing blood glucose levels (10) while also improving blood lipids, blood pressure, cardiovascular health, and well-being in patients with T2DM (11).

There is a consensus among many scholars that regular aerobic exercise training (AET) can improve blood glucose, blood lipids, blood pressure, and physical health in adults with T2DM (12, 13). With the advancement of exercise metabolism research, resistance training (RT) has become an important intervention to reduce glucose in conjunction with AET and some studies have demonstrated that combined training (CBT) exercise can improve lipid and blood glucose levels in patients with T2DM more than aerobic or resistance exercise (14), which is also effective in middle-aged and elderly patients with T2DM (15). As research continues, some scholars believe that moderate-intensity continuous training (MICT) requires a greater time commitment and has poor patient compliance, while high-intensity interval training (HIIT) requires less time and has a high compliance rate. Some studies have demonstrated that HIIT is equally effective in patients with T2DM (16), and can improve blood glucose and lipid levels in patients, but the findings are still controversial (17). Method training (MT) is a type of physical and mental guiding exercise based on traditional Chinese medicine (TCM) for health and rehabilitation and the basic theory of Chinese medicine, which is the treasure of Chinese culture and has a unique health effect. As a low-to-moderate intensity form of exercise, many studies in recent years have demonstrated that traditional Chinese medicine MT improves blood lipids and blood glucose in patients with T2DM (18). As a traditional Chinese fitness exercise, Taichi is a low-to-medium intensity exercise with the waist as the axis, and AET has the bones as the axis. The movements are soft and slow, are simple and easy to learn, and the level of exercise is low. The meta-analysis showed that Taichi helps improve blood glucose control, reduce body weight, regulate blood lipids, and improve the quality of life in patients with T2DM (19), which is consistent with previous analytical discussions but is still controversial. The results of previous studies suggest that MT can reduce blood glucose levels in patients, but the effect of the intervention on blood lipid levels may be influenced by age and the duration of intervention (20).

Among the five exercise interventions studied, the available evidence suggests that AET is the most applied treatment modality with good results in treating complications and improving physical fitness (21). The efficacy of RT is correlated to the intensity of the exercise (22). CBT is suggested to be the best way to control blood glucose compared to AET or RT (23). HIIT for T2DM is poorly documented, so its safety needs to be further verified (24). MT, a traditional exercise modality, has also shown some effectiveness in patients with T2DM. In various studies, the means of exercise intervention varied, and the results were controversial, and the majority of studies did not investigate the effects of different exercise modalities on middle-aged and elderly patients with T2DM. Given the different research results, this study applied a systematic evaluation and meta-analysis approach to further clarify the evidence on the effects of multiple exercise interventions on blood lipid and blood glucose levels and explore more effective interventions in middle-aged and elderly patients with T2DM.

## 2 Materials and methods

### 2.1 Inclusion and exclusion criteria

#### 2.1.1 Study type

Randomized controlled trial (RCT).

#### 2.1.2 Study subjects

Patients with T2DM; age >45 years; met the 1999 WHO diagnostic criteria for T2DM.

#### 2.1.3 Interventions

The experimental group had HIIT, AET, RT, CBT, and MT as treatments; the control group had conventional treatment or a blank control. All the interventions are clearly defined in **Table 1**.

**Table 1:** Definitions of exercise interventions and control group.

Interventions	Abbreviation	Definition
**Exercise therapy**	HIIT	HIIT is a training method in which people perform multiple exercises of a few seconds or minutes at a load intensity ≥ anaerobic threshold or maximal lactate homeostasis, and the arrangement between each exercise makes it insufficient for the practitioner to recover, i.e., rest or low-intensity exercise (25). The majority of trials used 20 to 40 sessions of power cycling or running with a duration of 60 seconds at 90% VO2max and a 60-second interval between groups.
	AET	AET is a continuous and repeated physical exercise. The main mode of energy supply is aerobic metabolism, such as jogging, healthy walking, swimming, cycling, and ball games (11). The exercise mode is walking, running, and cycling with an exercise intensity of 60% VO2max for 30 to 60 min.
	RT	RT is also called muscle strength training and aims to increase muscle mass, which can increase basal metabolic rate, improve skeletal muscle weight-bearing capacity, reduce cardiovascular risk factors, and decrease all-cause mortality (11). The training method is multiple repetitions of 75% to 85%1RM on machines.
	CBT	CBT exercise combines both aerobic and strength training. The exercise intensity is 30 min of 60%-70%1RM RT, and 30 min of 60%-80%HRmax walking, running, and cycling.
	MT	MT includes traditional sports, such as Taichi and Baduanjin (26). Exercise intensity of 60%-70%HRmax and duration of 40-60min moderate intensity training.
**Control group**	Conventional treatment	Using conventional treatment and care, such as medications, dietary interventions, and other non-exercise interventions, without additional heavy physical activity during the trial period,

#### 2.1.4 Outcome indicators

Blood glucose indexes: glycosylated hemoglobin (HbA1c), fasting blood glucose (FBG). Blood lipid indexes: total cholesterol (TC), triglycerides (TG), high-density lipoprotein (HDL), and low-density lipoprotein (LDL).

#### 2.1.5 Exclusion criteria

① Studies from which data could not be extracted; ② republished literature; ③ people with other specific diseases, such as muscle or joint disability, heart disease, and coronary artery disease and who are intolerant to exercise.

### 2.2 Search strategies

We searched databases, such as CNKI, VIP, WanFang Data, PubMed, Web of Science, The Cochrane Library, and Embase, to retrieve RCTs on the effects of exercise interventions on blood glucose and blood lipid levels in patients with T2DM. The search time frame was from the creation of the database to October 2022. Chinese literature was searched using subject terms such as”HIIT, aerobic exercise training, resistance training, combined training, Baduanjin, Taichi, exercise intervention, T2DM” and English literature was searched by subject terms such as “high-intensity interval training OR interval training OR aerobic exercise OR aerobic training OR oxygen sports OR resistance exercise OR combined training OR Exercise Therapy OR exercise OR training OR Baduanjin OR Taichi” AND “type 2 diabetes mellitus OR T2D OR T2DM”. Subject terms were freely combined.

### 2.3 Literature screening and data extraction

Two independent researchers (Yifan Zhang and Shijie Feng) conducted the literature screening and data extraction (which included: author, year, sample size, age, intervention, intervention period, outcome indicators, etc.), and other researchers intervened to negotiate if there was a dispute.

### 2.4 Risk of bias assessment of included studies

The risk of bias assessment of the included literature was performed by two independent researchers according to the Cochrane Systematic Reviews risk of bias assessment tool(ROB2) (27), which includes election bias (random sequence generation, allocation concealment), performance bias (blinding of participants and personnel), detection bias (blinding of outcome assessment), attrition bias (incomplete outcome data), reporting bias (selective reporting), and other bias (other sources of bias).

### 2.5 Statistical analysis

Review Manager 5.3 software was used to test the heterogeneity of the original studies with a direct comparison of two identical interventions. I2 ≤ 50% and P>0.1 indicated no significant heterogeneity between studies. I2≥50% and P ≤ 0.1 indicated greater heterogeneity between studies, and further analysis of the sources of heterogeneity was required. Stata 16.0 software was used for network meta-analysis and nodal analysis was used to test for inconsistencies. If P > 0.05, the difference between direct and indirect comparisons had no statistical significance, which indicated that the two results were consistent and were analyzed using the consistency model. Conversely, the inconsistency model was used. Next, a two-by-two comparison between different exercise interventions was performed. P < 0.05 indicated that the difference was statistically significant, and the surface under the cumulative ranking (SUCRA) method was used to rank multiple interventions for comparison. There needed to be at least three articles with the same exercise therapy before we ranked the effect of the intervention. SUCRA = 1 indicated that the intervention was absolutely effective, while SUCRA = 0 indicated that the intervention was absolutely ineffective (28).

## 3 Results

### 3.1 Literature screening process and results

**Figure 1:**
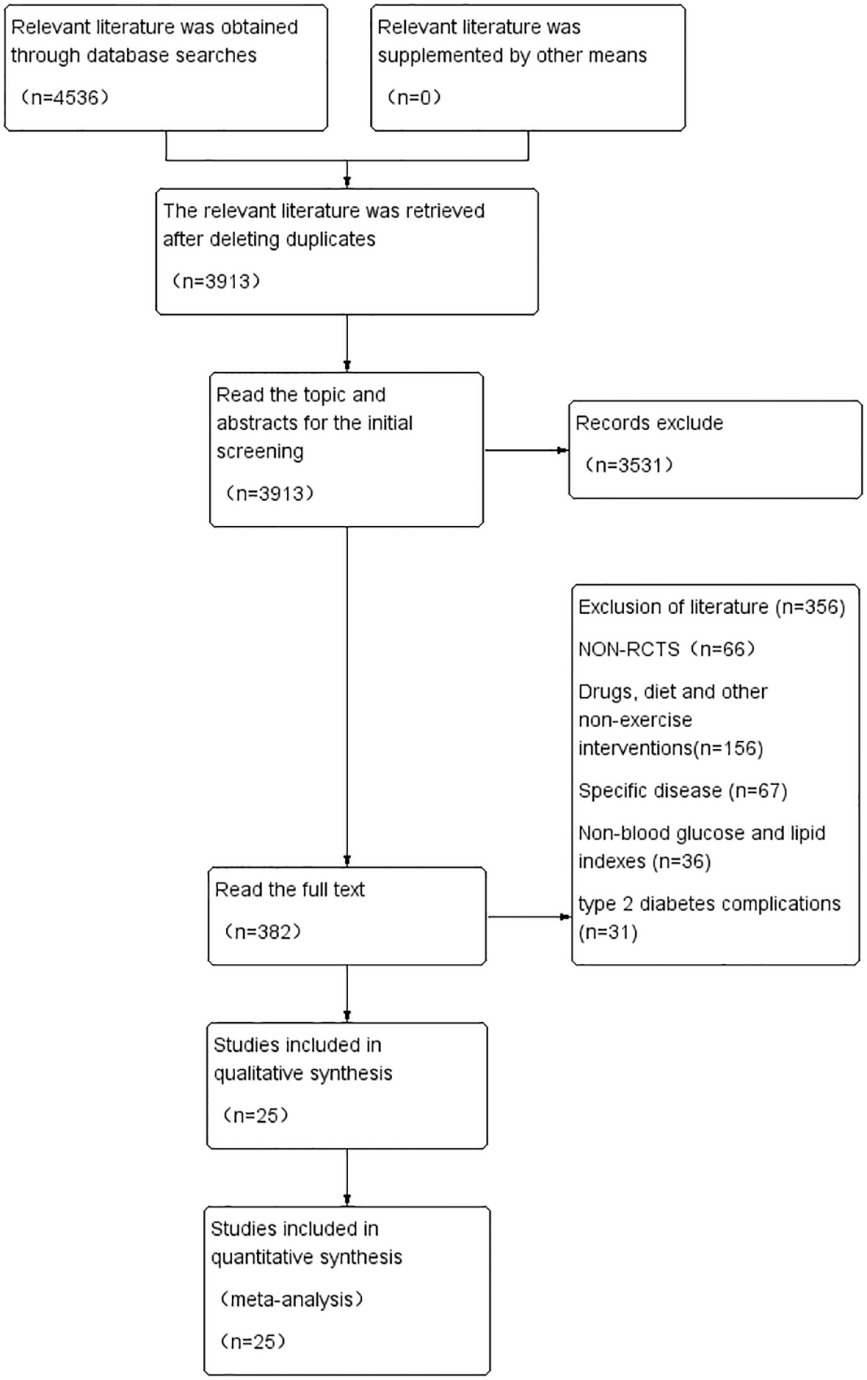
Flow chart and results of literature screening.

A total of 4,536 papers were obtained from the initial review, and after stratum-by-stratum screening, 25 papers with 1,711 patients with T2DM were finally selected. The literature screening process and results are shown in **Figure 1**.

### 3.2 Basic characteristics of the included studies and results of risk of bias assessment

**Figure 2:**
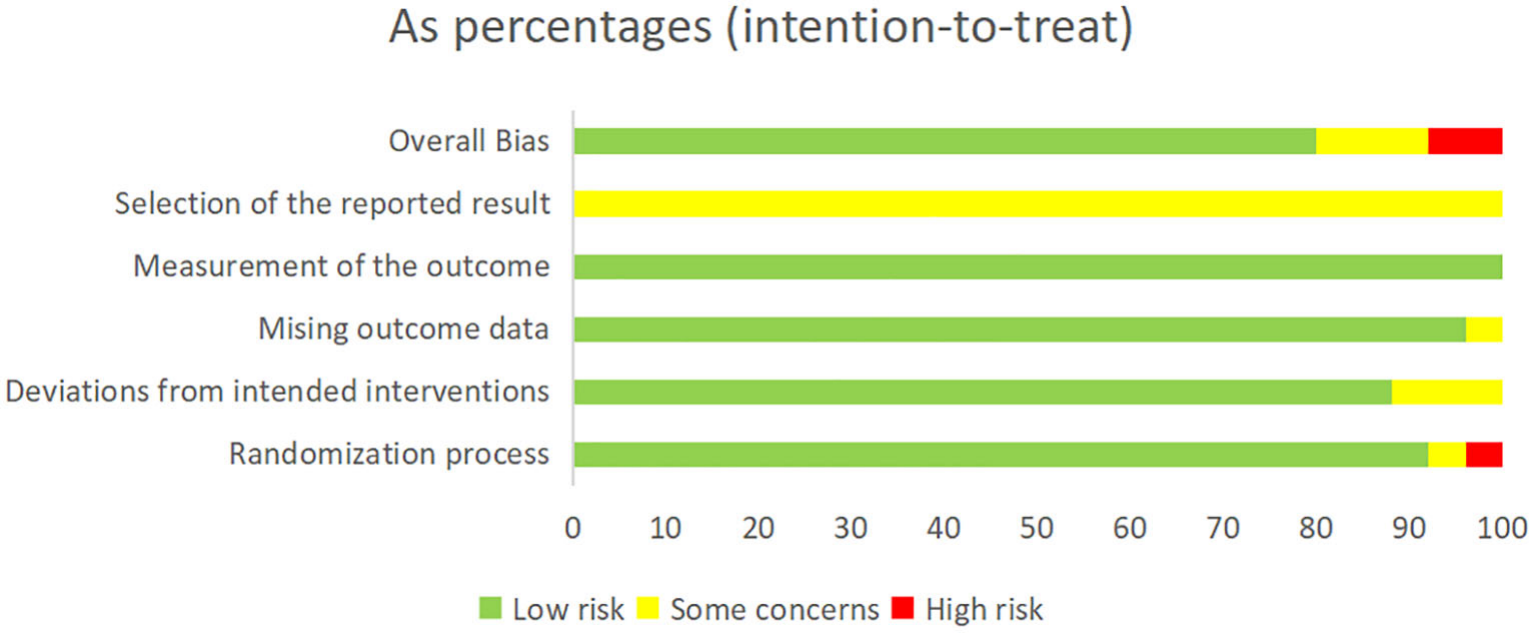
Summary of risk-of-bias assessment according to the revised Cochrane risk-of-bias tool for randomized trials.

**Figure 3:**
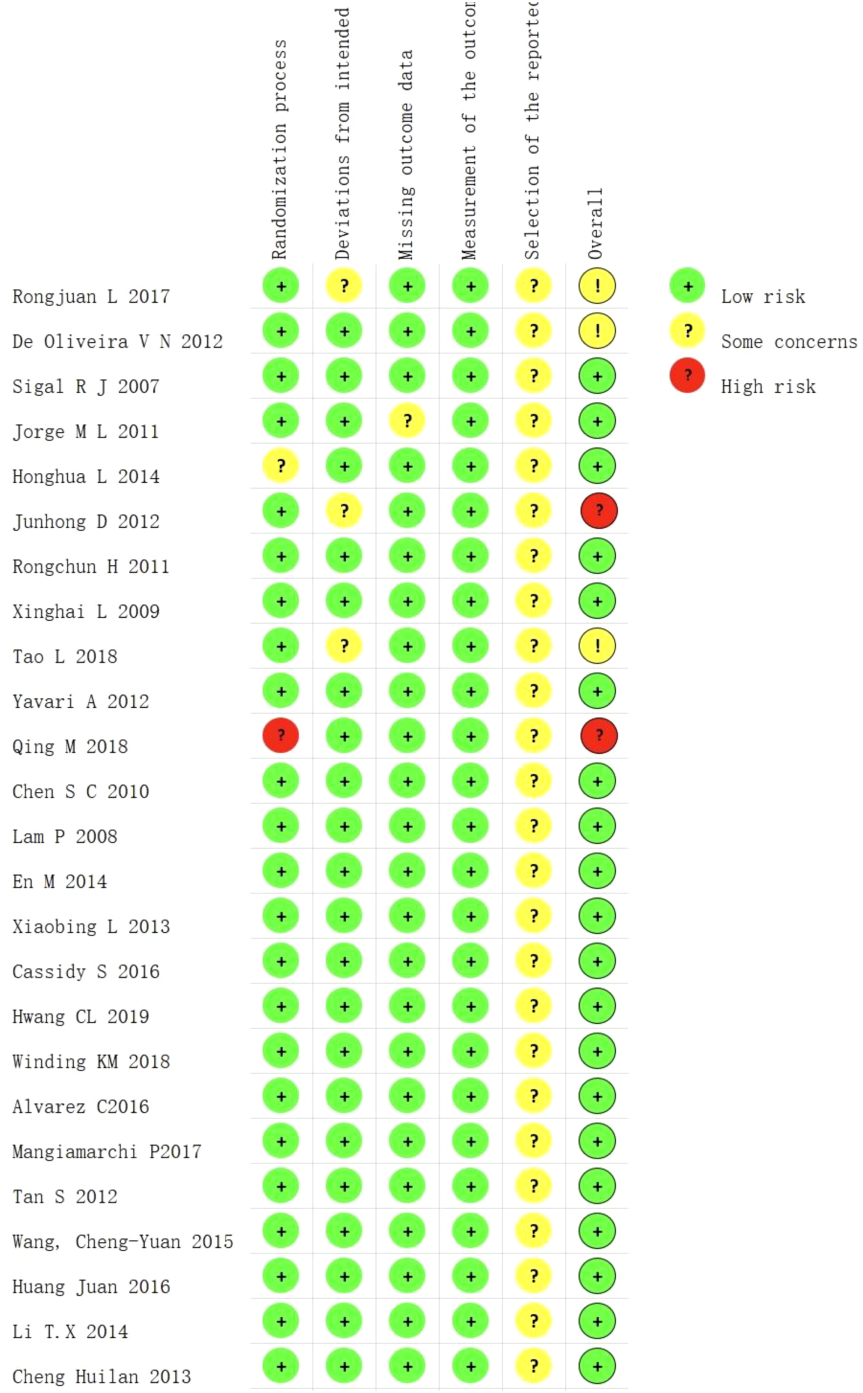
Risk of bias assessment according to the revised Cochrane risk-of-bias tool for randomized trials.

The basic characteristics of the included studies are shown in **Table 2**, and the results of the risk of bias assessment are shown in **Figures 2** and **3**. The risk of bias assessment rated 23/25 studies “low risk” or “having some concerns” and two studies were rated “high risk” due to a lack of information on the measurement of the outcome and missing outcome data.

**Table 2:** Basic characteristics of the included studies.

Included studies	Sample size (cases)	Age (T/C, years)	Intervention mean	Intervention period	Outcome indicators
	(T/C)				
Cassidy S (29), 2016	12/11	61 ± 9/59 ± 9	HIIT	12 weeks	①②③④
Hwang CL (2), 2019	18/16	65 ± 2/61 ± 2	HIIT	8 weeks	①②③④⑤⑥
Winding KM (30), 2018	13/7	54 ± 6/57 ± 7	HIIT	11 weeks	①②③④⑤⑥
Alvarez C (31), 2016	13/10	45.6 ± 3.1/43.1 ± 1.5	HIIT	16 weeks	③④⑤⑥
Mangiamarchi P (32), 2017	9/10	57.5 ± 5.93/54 ± 7.9	HIIT	12 weeks	③④⑤⑥
Li Rongjuan (33), 2017	18/18/18	55 years old and above	AET and RT	18 weeks	①②③④⑤⑥
de Oliveira VN (34), 2012	11/10/10/12	52.09 ± 8.71/54.10 ± 8.94/ 57.90 ± 9.82/53.42 ± 9.82	AET, RT, and CBT	12 weeks	①②③④⑤⑥
Tan S (35), 2012	15/10	65.9 ± 4.2/64.8 ± 6.8	CBT	6 months	①②③④⑤⑥
Sigal RJ (36), 2007	60/64/64/63	53.9 ± 6.6/54.7 ± 7.5/ 53.5 ± 7.3/54.8 ± 7.2	AET, RT, and CBT	6 months	①③⑤⑥
Jorge ML (37), 2011	12/12/12/12	52.09 ± 8.71/54.10 ± 8.94 /57.90 ± 9.82/53.42 ± 9.82	AET, RT, and CBT	12 weeks	①②③④⑤
Wang Chengyuan (38), 2015	20/30	61.3 ± 8.4/61.7 ± 6.9	MT	6 weeks	①②③④⑤⑥
Liu Honghua (39), 2014	20/20	57 ± 7/55 ± 9	MT	6 months	①
Duan JH (40), 2012	100/100	47 ± 7/45 ± 9	MT	8 weeks	①②④⑤
Huang Rongchun (41), 2011	30/30	57.8± 7.5/56.5 ± 6.9	MT	6 months	①②③④⑤⑥
Li Xinghai (42), 2009	40/39	57.8 ± 7.5/56.5 ± 6.9	MT	6 months	①②③④⑤⑥
Liu T (43), 2018	20/20	57.2 ± 5.4	MT	24 weeks	①②③④⑤⑥
Yavari A (44), 2012	15/15/15/15	48.2 ± 9.2/51.5 ± 6.3/ 50.9 ± 9.8/51.5 ± 8.5	AET, RT, and CBT	52 weeks	①②③④⑤⑥
Huang Juan (45), 2016	16/10	48 years old	CBT	8 weeks	①②
Li T.X (46), 2014	20/20/20/20	56.3 ± 7.01/55.63 ± 6.78/ 55.56 ± 6.59/56.28 ± 6.38	AET, RT, and CBT	12 weeks	①②③④⑤⑥
Cheng Huilan (47), 2013	30/30	69.5 ± 5.7	RT	9 weeks	①②③④⑤⑥
Meng Q (48), 2018	40/40/40	61.1 ± 7.6/61.4 ± 11.6/ 63.5 ± 9.2	AET and CBT	3 months	①②
Chen SC (49), 2010	56/48	59.1 ± 6.2/57.4 ± 5.8	MT	12 weeks	①②③④⑤
Lam P (50), 2008	28/25	63.2 ± 8.6/60.7 ± 12.2	MT	3 months	①③④
Meng E (51), 2014	100/100	68.4 ± 3.2	MT	3 months	①②③④⑤⑥
Li X. B (52), 2013	30/30	50.7 ± 7.3	MT	8 weeks	②

① glycosylated hemoglobin; ② fasting blood glucose; ③ triglycerides; ④ total cholesterol; ⑤ high-density lipoprotein; ⑥ low-density lipoprotein; AET, aerobic exercise training; RT, resistance training; CBT, combined training; MT, method training; HIIT, high-intensity interval training.

### 3.3 Network diagram and consistency analysis

#### 3.3.1 Network diagram

**Figure 4:**
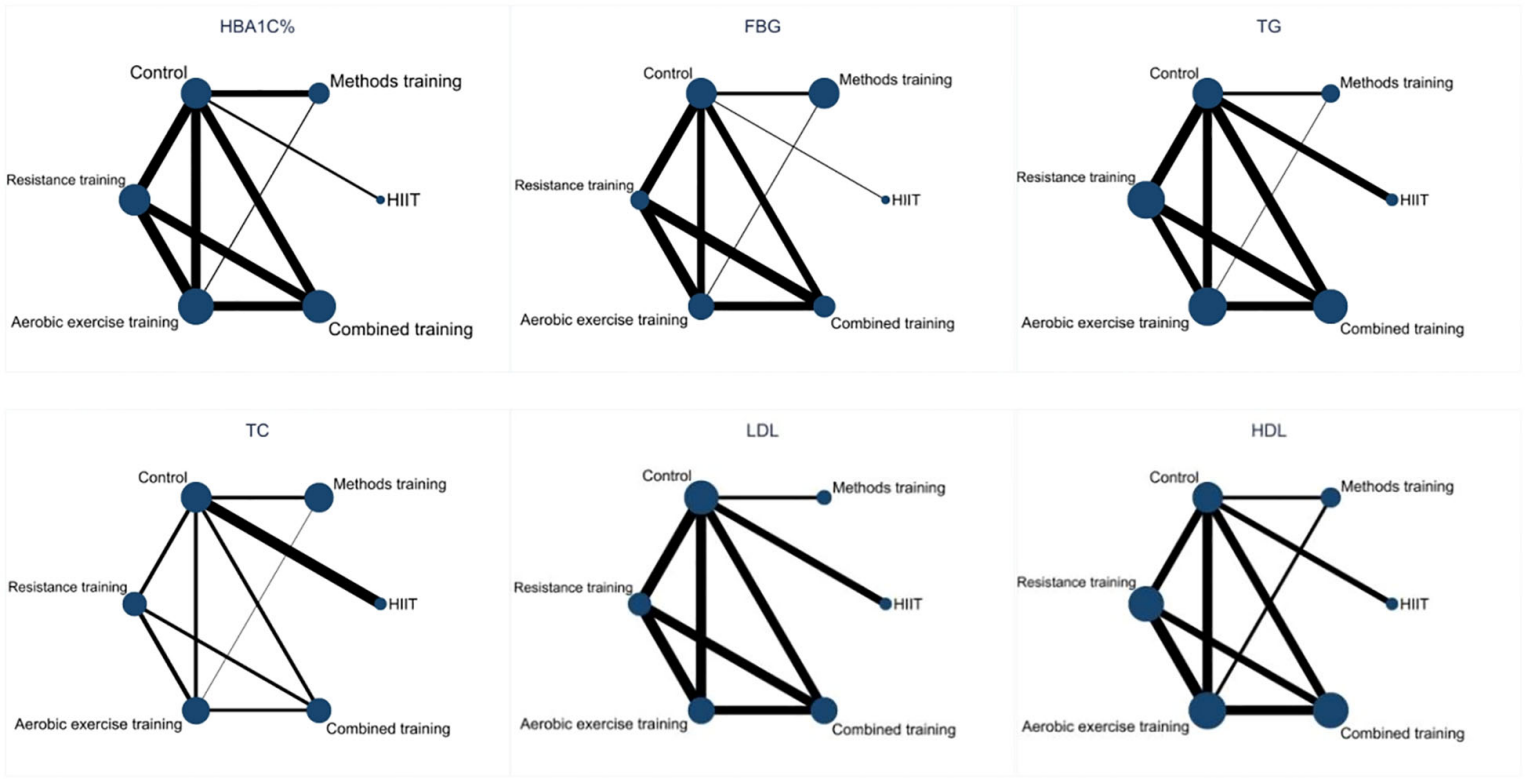
Network diagram of the effects of five exercise interventions on patients with T2DM.

The network relationships between the interventions are shown in **Figure 4**, involving five exercise interventions. The dots in the figure represent the interventions.

The solid lines between the dots represent direct comparisons between the interventions, and the thicker the solid line, the greater the number of studies.

#### 3.3.2 Consistency analysis

Inconsistency tests were performed on the 25 included RCTs using nodal analysis. The nodal analysis model showed the HbA1c (P=0.8231), FBG (P=0.9219), TG (P=0.9433), TC (P=0.9272), HDL (P=0.9871), and LDL results (P=0.9359). All were >0.05, indicating that the direct comparison of the six indicators of blood glucose and the indirect comparison results were consistent. Therefore, the consistency model can be used for the analysis.

#### 3.3.3 Sensitivity analysis

Heterogeneity tests were conducted separately on the original studies with direct comparisons between two identical interventions under each effect indicator. The results showed that when HbA1c was used as an indicator, the heterogeneity between the studies of Rongjuan et al. and Jorge et al. was high (I^2^ = 73%, p<0.001), presumably because of gender differences. When FBG was used as an indicator, the heterogeneity between the studies of Rongjuan et al., de Oliveira, and Jorge et al. was high (I^2^ = 91%, p<0.001, presumably because of the different intervention cycles and intervention intensities. When TG was used as an indicator, the heterogeneity between the studies of Alvarez et al. and Cheng et al. was high (I^2^ = 89%, P<0.001), presumably because of the large differences in the time of intervention. When TC was used as an indicator, the heterogeneity between the studies of Rongjuan et al. and Cheng et al. was high (I^2^ = 91%, P<0.001), presumably due to differences in intervention modality and gender.

### 3.4 Results of the network meta-analysis

#### 3.4.1 Effect of different exercise interventions on HbA1c

The results of the network meta-analysis showed that MT [MD=-0.69, 95%CI (-0.98,-0.40), P<0.05], CBT [MD=0.63, 95%CI (0.39,0.87), P<0.05], HIIT [MD=-0.57,95%CI (-1.06,-0.07), P<0.05], AET [MD=0.51, 95%CI (0.26,0.75), P<0.05], and RT [MD=0.29, 95%CI (0.03,0.55), P<0.05] were superior to the control group. Indirect comparisons showed that both MT and CBT were superior to RT, and the differences between the other exercise methods were not statistically significant (P > 0.05) (**Table 3**). The ranking of SUCRA probabilities was as follows: MT (SUCRA=82.0), CBT (SUCRA=76.0), HIIT (SUCRA=63.3), AET (SUCRA=53.5), and RT (SUCRA=24.6), suggesting that MT may have the best effect on HbA1c (**Table 4**).

**Table 3:** Two-by-two comparison results of five exercise interventions on HbA1c and FBG in patients with T2DM [MD (95% CI)].

HIIT	0.39 (-0.56,1.34)	1.08 (0.24,1.92)	0.62 (-0.32,1.55)	0.55 (-0.37,1.47)	0.43 (-0.49,1.36)
0.12 (-0.45,0.70)	MT	0.69 (0.24,1.13)	0.23 (-0.37,0.82)	0.16 (-0.40,0.72)	0.04 (-0.54,0.63)
-0.57 (-1.06,-0.07)	-0.69 (-0.98,-0.40)	Conventional treatment	-0.46 (-0.87,-0.05)	-0.53 (-0.91,-0.14)	-0.64 (-1.04,-0.25)
-0.28 (-0.84,0.28)	-0.40 (-0.79,-0.02)	0.29 (0.02,0.55)	RT	-0.07 (-0.50,0.36)	-0.18 (-0.63,0.26)
-0.06 (-0.61,0.49)	-0.19 (-0.55,0.18)	0.51 (0.26,0.75)	0.22 (-0.06,0.49)	AET	-0.12 (-0.53,0.30)
0.07 (-0.48,0.62)	-0.06 (-0.43,0.31)	0.63 (0.39,0.87)	0.35 (0.07,0.63)	0.13 (-0.13,0.39)	CBT

The bottom left is the result of the HbA1c two-by-two comparison, and the top right is the result of the FBG two-by-two comparison.

**Table 4:** Probability ranking results of the effects of the five exercise interventions on patients with T2DM (SUCRA values).

Intervention	HbA1c score	FBG score	TG score	TC score	HDL score	LDL score
HIIT	63.3	87.4	82.0	25.2	91.8	13.1
MT	82.0	65.4	91.3	58.6	85.5	88.2
RT	24.6	37.7	31.5	90.5	17.7	77.7
AET	53.5	46.4	44.0	69.2	47.9	54.7
CBT	76.0	62.6	51.0	47.7	50.2	44.8
Conventional treatment	0.6	0.5	0.2	8.9	6.9	21.5

#### 3.4.2 Effect of different exercise interventions on FBG

The results of the network meta-analysis showed that HIIT [MD=1.08, 95%CI (0.24,1.92), P<0.05], MT [MD=0.69, 95%CI (0.24,1.13), P<0.05], and CBT [MD=-0.64, 95%CI (-1.04,-0.25), P<0.05], AET [MD=-0.53, 95%CI (-0.91,-0.14), P<0.05], and RT [MD=-0.46, 95%CI (-0.87,-0.05), P<0.05] were superior to the control group. The results of the indirect comparison showed that the differences between the exercise methods were not statistically significant (P > 0.05) (**Table 3**). The ranking of SUCRA probabilities was as follows: HIIT (SUCRA=87.4), MT (SUCRA=65.4), CBT (SUCRA=62.6), AET (SUCRA=46.4), and RT (SUCRA=37.7), suggesting that HIIT interventions may have the best effect on FBG (**Table 4**).

#### 3.4.3 Effect of different exercise interventions on TG

The results of the network meta-analysis showed that MT [MD=-1.00, 95%CI (-1.41,-0.59), P<0.05], HIIT [MD=-0.90, 95%CI (-1.51,-0.28), P<0.05], RT [MD=0.37, 95%CI (0.07,0.66), P< 0.05], AET [MD=0.45, 95% CI (0.16,0.75), P<0.05], and CBT [MD=0.50, 95% CI (0.19,0.81), P<0.05] were all superior to the control group. Indirect comparisons showed that MT was superior to RT, AET, and CBT, and the differences between the other exercise methods were not statistically significant (P > 0.05) (**Table 5**). The ranking of SUCRA probabilities was as follows: MT (SUCRA=91.3), HIIT (SUCRA=82.0), CBT (SUCRA=51.0), AET (SUCRA=44.0), and RT (SUCRA=31.5), suggesting that MT interventions may have the best effect on TG (**Table 4**).

**Table 5:** Two-by-two comparison results of five exercise interventions on TG and TC in patients with T2DM [MD (95% CI)].

HIIT	-0.48 (-1.54,0.59)	0.14 (-0.72,1.00)	-0.86 (-1.89,0.16)	-0.60 (-1.63,0.42)	-0.37 (-1.41,0.68)
0.10 (-0.64,0.84)	MT	0.62 (-0.00,1.24)	-0.39 (-1.20,0.43)	-0.13 (-0.92,0.66)	0.11 (-0.73,0.94)
-0.90 (-1.51,-0.28)	-1.00 (-1.41,-0.59)	Conventional treatment	-1.00 (-1.57,-0.44)	-0.75 (-1.31,-0.19)	-0.51 (-1.10,0.08)
-0.53 (-1.21,0.15)	-0.63 (-1.13,-0.14)	0.37 (0.07,0.66)	RT	0.26 (-0.33,0.84)	0.50 (-0.12,1.11)
-0.44 (-1.12,0.24)	-0.55 (-1.03,-0.07)	0.45 (0.16,0.75)	0.09 (-0.22,0.39)	AET	0.24 (-0.38,0.86)
-0.40 (-1.09,0.29)	-0.50 (-1.01,-0.00)	0.50 (0.19,0.81)	0.13 (-0.19,0.45)	0.05 (-0.27,0.36)	CBT

The lower left is the result of the two-two comparison of TG, and the upper right is the result of the two-two comparison of TC.

#### 3.4.4 Effect of different exercise interventions on TC

Network meta-analysis showed that RT [MD=-1.00, 95%CI (-1.57,-0.44), P<0.05], AET [MD=-0.75, 95%CI (-1.31,-0.19), P<0.05] were superior to the control group, while there was no evidence that HIIT [MD=0.14, 95%CI (-0.72,1.00), P>0.05], CBT [MD=-0.51, 95%CI (-1.10,0.08), P>0.05] and MT [MD=0.62,95%CI (-0.00,1.24), P>0.05], were more effective in treating TC than the control group. Indirect comparisons showed no statistically significant differences (P > 0.05) between the exercise modalities when compared with each other (**Table 5**). The ranking of SUCRA probabilities was as follows: RT (SUCRA=90.5), AET (SUCRA=69.2), MT (SUCRA=58.6), CBT (SUCRA=47.7), and HIIT (SUCRA=25.2), suggesting that the RT intervention may have the best effect on TC (**Table 4**).

#### 3.4.5 Effect of different exercise interventions on HDL

The results of the network meta-analysis showed that HIIT [MD=1.02, 95%CI (0.28,1.76), P<0.05], MT [MD=0.82, 95%CI (0.41,1.23), P<0.05], aerobic training [MD=-0.30, 95%CI (-0.60,1.76), P<0.05] and combined exercise training [MD=-0.32, 95%CI (-0.63,-0.01), P<0.05] were superior to the control group, while there was no evidence supporting that RT [MD=-0.09, 95%CI (-0.38,0.21), P>0.05] was superior to the control group in treating HDL. Indirect comparisons showed that HIIT was superior to RT. MT was superior to RT, AET, and CBT, and the differences between the other exercise methods were not statistically significant (P > 0.05) (**Table 6**). The ranking of SUCRA probabilities was as follows: HIIT (SUCRA = 91.8), MT (SUCRA = 85.5), CBT (SUCRA=50.2), AET (SUCRA=47.9), and RT (SUCRA=17.7), suggesting that HIIT interventions may have the best effect on HDL (**Table 4**).

**Table 6:** Two-by-two comparison results of five exercise interventions on HDL versus LDL in patients with T2DM [MD (95% CI)].

HIIT	-0.52 (-1.11,0.07)	-0.17 (-0.67,0.33)	-0.41 (-0.96,0.14)	-0.31 (-0.86,0.24)	-0.27 (-0.82,0.28)
0.20 (-0.64,1.04)	MT	0.35 (0.03,0.67)	0.11 (-0.27,0.49)	0.21 (-0.18,0.60)	0.25 (-0.14,0.64)
1.02 (0.28,1.76)	0.82 (0.41,1.23)	Conventional treatment	-0.24 (-0.46,-0.02)	-0.14 (-0.37,0.08)	-0.10 (-0.33,0.12)
0.94 (0.14,1.74)	0.73 (0.24,1.23)	-0.09 (-0.38,0.21)	RT	0.10 (-0.13,0.32)	0.14 (-0.09,0.37)
0.72 (-0.07,1.51)	0.52 (0.04,0.99)	-0.30 (-0.60,-0.01)	-0.22 (-0.52,0.09)	AET	0.04 (-0.19,0.28)
0.70 (-0.10,1.50)	0.50 (0.00,1.00)	-0.32 (-0.63,-0.01)	-0.24 (-0.56,0.09)	-0.02 (-0.34,0.30)	CBT

The HDL two-by-two comparison results are shown in the lower left, and the LDL two-by-two comparison results are shown in the upper right.

#### 3.4.6 Effect of different exercise interventions on LDL

Network meta-analysis showed that MT [MD=0.35, 95%CI (0.03,0.67), P<0.05] and RT [MD=-0.24, 95%CI (-0.46,-0.02), P<0.05] were superior to the control group, and there was no evidence that HIIT [MD=-0.17, 95%CI (-0.67, 0.33), P>0.05], CBT [MD=-0.10, 95%CI (-0.33,0.12), P>0.05], and AET [MD=-0.14, 95%CI (-0.37,0.08), P>0.05] were better than the control group in treating LDL. Indirect comparisons showed that the differences between exercise methods were not statistically significant (P > 0.05) (**Table 6**). The ranking of SUCRA probabilities was as follows: MT (SUCRA = 88.2), RT (SUCRA = 77.7), AET (SUCRA = 54.7), CBT (SUCRA = 44.8), and HIIT (SUCRA=13.1), suggesting that MT may be the most effective intervention for LDL (**Table 4**).

## 4 Discussion

### 4.1 Effects on glucose metabolism in middle-aged and elderly patients with T2DM

The pathophysiology of T2DM is characterized by a decreased ability to regulate glucose metabolism, which is accompanied by a functional defect in pancreatic beta cells, resulting in insufficient insulin secretion. High glucose toxicity can cause islet beta cell dysfunction or even inactivation, which leads to islet beta cell dormancy and reduces insulin secretion by 70% or more. Exercise interventions can significantly enhance pancreatic beta cell function, increase insulin secretion, and decrease blood glucose levels in patients (53). In recent years, healthcare professionals have applied exercise interventions as an adjunct to the prevention, treatment, and rehabilitation stages of diabetes, chronic obstructive pulmonary disease, hypertension, and sub-health states, and these have achieved certain results. There is clinical evidence suggesting that exercise interventions combined with conventional therapeutic options can effectively reduce glucose and lipid metabolism levels in patients with diabetes, with better results than conventional therapeutic options alone (30–34, 54, 55).

HbA1c is recognized worldwide as a key indicator of blood glucose control in patients with T2DM, and HbA1c monitoring is important for disease control and prediction of complications in diabetic patients. The results of this review suggest that all five exercise interventions can reduce HbA1c and that MT may have the best effect on HbA1c. The results of the meta-analysis of the effects on HbA1c in this article are generally consistent with the results of previous studies (56). Furthermore, a study using meta-analysis conducted a comprehensive analysis of the effects of TCM MT on patients with T2DM, and the results suggest that an intervention period of 2–3 months with 30–60 min of activity each time was able to reduce the glycosylated hemoglobin index of the subjects (57). Another study revealed that method training 3–6 times a week will provide the best recovery results in subjects after 6 months (58). This evidence suggests that MT is better than other exercises in increasing plasma volume, improving the skeletal muscle uptake and utilization of blood glucose, accelerating glucose clearance, and improving HbA1c levels in middle-aged and elderly patients with T2DM (30, 59), but may be influenced by the cycle of intervention. Evidence suggests that MT mainly affects pancreatic function directly through breathing exercises, promoting beta cell secretion (41) and increasing the activity of insulin receptors, thus lowering patients’ blood glucose levels, and consequently improving their physical and mental status and quality of life (60). Intervention cycles and modalities were not standardized among the training methods included in this study, and the findings should be explored further in the future with sub-group analysis.

Regarding the effect on fasting glucose in patients with T2DM, the results of this review suggest that all five exercise modalities can reduce FBG levels, and the HIIT intervention was the most effective for FBG. Physical training can effectively improve insulin sensitivity and glucose-carrying protein activity (GLUT4), and reduce postprandial blood fat and systemic inflammatory response, controlling blood glucose levels. In addition, when the exercise stops, the decrease in the body’s muscle glycogen content will increase the rate of glycogen synthesis, promoting the absorption of glucose by the liver and muscles and replenishing the glycogen consumed by the body during exercise (61), which is very important to prevent a variety of complications caused by disorders of glucose metabolism in diabetic patients. By tracking and analyzing the original literature, HIIT training consisted of 1–4 min of training at more than 90% of maximum heart rate and active recovery at approximately 70% of maximum heart rate, with an exercise lasting 25–35 min. Treadmill training 3–5 times a week may significantly improve fasting blood glucose levels in subjects within 8–12 weeks. HIIT training requires dedicated guidance, real-time detection of changes in heart rate and pulse rate, and control of exercise intensity.

### 4.2 Effects on lipid metabolism in middle-aged and elderly patients with T2DM

Regarding the effect on cholesterol in patients with T2DM, the results of this study suggest that RT and AET can reduce TC levels, and RT intervention may be the most effective for TC, while there was no evidence that HIIT, CBT, and MT are superior to the control group for TC. Elevated lipids, especially TC and LDL-C, can cause a significant increase in fasting glucose levels in patients with T2DM, but with a decrease in lipid control, their fasting glucose will also decrease (62). Hyperlipidemia significantly increases the risk of complications such as cardiovascular disease, diabetic nephropathy, and diabetic retinopathy in patients with T2DM. These intermediate and long-term complications of diabetes are often the main cause of death and disability in diabetic patients. Evidence suggests that after 18 weeks of exercise intervention, RT was more advantageous than AET in improving TC and LDL in subjects, which is consistent with the results of this study. RT leads to an improvement in the morphological structure and physiological function of the muscle, which promotes the gene expression of the transporters of glucose and accelerates the uptake of glucose by the cells, resulting in lower blood glucose and lipid levels in the body (33). Resistance exercise is based on the main joint and muscle circulation exercises of the whole body. Exercise intensity was 50%–80% of the maximum weight, movement frequency was 8–12 times per movement, and exercise frequency was 3–5 times per week, and included 10 mins of preparation activities and 5 min of stretching activities. In addition, resistance exercise requires personal guidance and real-time testing of the exercise process.

Regarding the effect on TG in patients with T2DM, the results of this review found that all five exercise modalities can lower TG levels and MT interventions may have the best effect on TG. As one of the low-to-medium-intensity forms of exercise, MT is mainly based on aerobic metabolism for energy supply. Long-term exercise enhances the body’s utilization of fat, regulates the metabolism of lipoprotein in the body (63), reduces the body’s fat content, increases energy expenditure, and reduces glycogen reserves in the liver and muscles (64), thus achieving weight and lipid control. The reduction of cholesterol and TG levels in the blood can largely alleviate and effectively prevent the deposition of cholesterol in the walls of arteries, thus achieving the effect of reducing, preventing, and treating atherosclerosis and reducing the incidence of cerebrovascular diseases (65). Some studies have demonstrated that MT is more effective than other exercises in improving blood glucose metrics, physical health, and the body’s antioxidant and anti-inflammatory activation in patients with T2DM. In addition, MT can reduce serum high-sensitivity C-reactive protein (HsCRP) and malondialdehyde (MDA) levels to reduce the risk of certain oxidative and atherosclerotic complications (19, 49). However, it has also been shown that Taichi does not significantly improve TG concentrations in diabetic patients (20), which is a somewhat controversial issue and still needs to be further explored in depth.

Regarding the effect on HDL in patients with T2DM, the results of this review suggest that HIIT, MT, AET, and CBT can improve HDL levels, and HIIT interventions may be the best for HDL levels. Exercise interventions can increase LPL activity, promote muscle uptake and utilization of more fatty acids and cholesterol, and accelerate the transfer of cholesterol and phospholipids to HDL, resulting in higher HDL levels (65). Some studies have shown that HIIT has also shown some effectiveness in reducing body weight and skinfold thickness in subjects (31, 32). For the effect on LDL, the results of this study showed that MT and RT both significantly improved LDL levels, and MT interventions may have the best effect on LDL, which is consistent with the results of previous studies (38, 66).

## 5 Conclusion

According to the study findings, MT appears to be the optimal choice to improve HbA1c, TG levels, and LDL while HIIT improves FBG and HDL levels. RT exercise appears to be the optimal exercise for lowering cholesterol levels. Careful consideration of the intensity, frequency, and duration of exercise, based on an individual’s physical and mental health, is important to optimize treatment outcomes. The few studies included in this systematic assessment conducted safe testing and adverse effect observation of MT, and their results suggest that MT is safe and can be further promoted under the guidance of professionals.

## 6 Limitations and shortcomings

① The majority of included studies did not report specific allocation concealment, blinding, and attrition, which may have resulted in some selection and measurement bias. ② The effectiveness of exercise therapy interventions in patients of different ages and duration of disease still needs to be further investigated. ③ There are few studies of blank control groups in randomized intervention trials on HIIT in patients with diabetes, so the overall number of included studies may be insufficient, and the results may require further validation, ④ Intervention cycles were not standardized among the different intervention modalities, and the effects of different intervention cycles on patients with T2DM need further validation.

## Data Availability

The original contributions presented in the study are included in the article/supplementary material. Further inquiries can be directed to the corresponding authors.

## References

[1] Sampath Kumar A Maiya AG Shastry BA Vaishali K Ravishankar N Hazari A . Exercise and insulin resistance in type 2 diabetes mellitus: A systematic review and meta-analysis. Ann Phys Rehabil Med. (2019) 62:98–103. doi: 10.1016/j.rehab.2018.11.001 30553010

[2] Hwang CL Lim J Yoo JK Kim HK Hwang MH Handberg EM . Effect of all-extremity high-intensity interval training vs. moderate-intensity continuous training on aerobic fitness in middle-aged and older adults with type 2 diabetes: A randomized controlled trial. Exp Gerontol. (2019) 116:46–53. doi: 10.1016/j.exger.2018.12.013 30576716 PMC6404965

[3] Li Y Teng D Shi X Qin G Qin Y Quan H . Prevalence of diabetes recorded in mainland China using 2018 diagnostic criteria from the American Diabetes Association: national cross-sectional study. BMJ. (2020) 369:m997. doi: 10.1136/bmj.m997 32345662 PMC7186854

[4] Willer CJ Schmidt EM Sengupta S Peloso GM Gustafsson S Kanoni S . Discovery and refinement of loci associated with lipid levels. Nat Genet. (2013) 45:1274–83. doi: 10.1038/ng.2797 PMC3838666 24097068

[5] Rasooly RS Akolkar B Spain LM Guill MH Del Vecchio CT Carroll LE . The National Institute of Diabetes and Digestive and Kidney Diseases Central Repositories: a valuable resource for nephrology research. Clin J Am Soc Nephrol. (2015) 10:710–5. doi: 10.2215/CJN.06570714 PMC4386252 25376765

[6] Mathers CD Loncar D . Projections of global mortality and burden of disease from 2002 to 2030. PloS Med. (2006) 3:e442. doi: 10.1371/journal.pmed.0030442 17132052 PMC1664601

[7] Clinical Guidelines for Prevention and Treatment of Type 2 Diabetes Mellitus in the Elderly in China (2022 Edition) drafting group . Clinical guidelines for prevention and treatment of type 2 diabetes mellitus in the elderly in China (2022 edition). Chin J Diabetes. (2022) 30:2–51. doi: 10.3969/j.issn.1006-6187.2022.01.002

[8] Fan K Yang X Zhu L . Research progress of exercise therapy in Type2 diabetic rats in recent 10 years. Contemp Sports Technol. (2017) 7:19–23. doi: 10.16655/j.cnki.2095-2813.2017.25.019

[9] Zou L Sasaki JE Wei GX Huang T Yeung AS Neto OB . Effects of mind^-^Body exercises (Tai chi/yoga) on heart rate variability parameters and perceived stress: A systematic review with meta-analysis of randomized controlled trials. J Clin Med. (2018) 7:404. doi: 10.3390/jcm7110404 30384420 PMC6262541

[10] Yang Q Huang R . Effects of baduanjin on psychology and life quality of type 2 diabetic patients accompanied with depression. Chin Med Mod Distance Educ China. (2017) 15:52–4. doi: 10.3969/j.issn.1672

[11] Zou D Zhang Z Ji L . Consensus of chinese experts on the remission of type 2 diabetes mellitus. Chin Gen Pract. (2021) 24:4037–48. doi: 10.12114/j.issn.1007-9572.2021.01.105

[12] Kanaley JA Colberg SR Corcoran MH Malin SK Rodriguez NR Crespo CJ . Exercise/physical activity in individuals with type 2 diabetes: A consensus statement from the american college of sports medicine. Med Sci Sports Exerc. (2022) 54:353–68. doi: 10.1249/MSS.0000000000002800 PMC8802999 35029593

[13] Gu Qi . Effects of aerobic exercise combined with different resistance training on the metabolism of blood glucose and blood lipid among elderly T2DM patients. J Xi’an Phys Educ Univ. (2021) 38:735–40. doi: 10.16063/j.cnki.issn1001-747x.2021.06.013

[14] Chen Y Zhang S Yu Z Pan L Zhang W . Effect of exercise on blood lipid for patients with type 2 diabetes: A network meta-analysis. Chin J Rehabil Theory Pract. (2019) 25:849–58. doi: 10.3969/j.issn.1006?9771.2019.07.022

[15] Xu Y Jing Q Zhao C . Effects of aerobic combined resistance exercise on oxidative stress and glycolipid metabolism in elderly patients with type 2 diabetes mellitus. Chin J Gerontol. (2019) 34:591–3. doi: 10.3969/j.issn.1005-9202.2019.03.029

[16] da Silva DE Grande AJ Roever L Tse G Liu T Biondi-Zoccai G . High-intensity interval training in patients with type 2 diabetes mellitus: a systematic review. Curr Atheroscler Rep. (2019) 21:8. doi: 10.1007/s11883-019-0767-9 30712240

[17] Chen B Guo J . Effects of high intensity interval training on type2 diabetes mellitus: a meta-analysis. Chin J Rehabil Theory Pract. (2018) 24:353–62. doi: 10.3969/j.issn.1006-9771.2018.03.020

[18] Zhang J Song C Gao C Zhang L . Meta analysis of clinical effects of TCM traditional exercise in the treatment for primary hypertension. Western J Traditional Chin Med. (2021) 34:79–84. doi: 10.12174/j.issn.2096-9600.2021.05.21

[19] Li H Qiu Y Tie Y . Effect of Chen’s TaiChi on blood biochemical indexes and cardiopulmonary function in elderly patients with type 2 diabetes. Chin J Gerontol. (2015) 5):1293–4. doi: 10.3969/j.issn.1005-9202.2015.05.067

[20] Su Z Hong P . Meta-analysis of effects of Tai Chi exercise on glycoMetabolism to the pathoglycemia populations. J Shaanxi Normal University(Natural Sci Edition). (2019) 47:38–47 + 125. doi: 10.15983/j.cnki.jsnu.2019.03.235

[21] Yang Z Scott CA Mao C Tang J Farmer AJ . Resistance exercise versus aerobic exercise for type 2 diabetes: a systematic review and meta-analysis. Sports Med. (2014) 44:487–99. doi: 10.1007/s40279-013-0128-8 24297743

[22] Al-Mhanna SB Batrakoulis A Wan Ghazali WS Mohamed M Aldayel A Alhussain MH . Effects of combined aerobic and resistance training on glycemic control, blood pressure, inflammation, cardiorespiratory fitness and quality of life in patients with type 2 diabetes and overweight/obesity: a systematic review and meta-analysis. PeerJ. (2024) 12:e17525. doi: 10.7717/peerj.17525 38887616 PMC11182026

[23] Church TS Blair SN Cocreham S Johannsen N Johnson W Kramer K . Effects of aerobic and resistance training on hemoglobin A1c levels in patients with type 2 diabetes: a randomized controlled trial. JAMA. (2010) 304:2253–62. doi: 10.1001/jama.2010.1710 PMC3174102 21098771

[24] Zhang M Cheng W Ma H . Analysis on the hotspot and content of exercise therapy for the treatment of type 2 diabetes mellitus in China—based on visualization research of scientific knowledge map. Chin J Diabetes. (2021) 29:104–11. doi: 10.3969/j.issn.1006-6187.2021.02.006

[25] Li Y . Effect of high-intensity interval training on different training populations. China Sport Sci. (2015) 8):59–75,96. doi: 10.16469/j.css.201508009

[26] Yang J . Comments and suggestions on the classification and terminology of exercise therapy. Chin J Rehabil Med. (2005) 20:371–3. doi: 10.3969/j.issn.1001-1242.2005.05.021

[27] Cumpston M Li T Page MJ Chandler J Welch VA Higgins JP . Updated guidance for trusted systematic reviews: a new edition of the Cochrane Handbook for Systematic Reviews of Interventions. Cochrane Database Syst Rev. (2019) 10:ED000142. doi: 10.1002/14651858.ED000142 31643080 PMC10284251

[28] Yi Y Zhang W Liu X Zhang J Zhu D Lv Q . Result interpretation of network meta-analysis. Chin J Evidence-Based Med. (2015) 15:103–9. doi: 10.7507/1672-2531.20140263

[29] Cassidy S Thoma C Hallsworth K Parikh J Hollingsworth KG Taylor R . High intensity intermittent exerciseimproves cardiac structure and function and reduces liver fat in patients with type 2 diabetes: a randomised controlled trial. Diabetologia: Clin Exp Diabetes Metab = Organ Eur Assoc Study Diabetes (EASD). (2016) 59:56–66. doi: 10.1007/s00125-015-3741-2 PMC4670457 26350611

[30] Winding KM Munch GW Iepsen UW Van Hall G Pedersen BK Mortensen SP . The effect on glycaemic control of low-volume high-intensity interval training versus endurance training in individuals with type 2 diabetes. Diabetes Obes Metab. (2018) 20:1131–9. doi: 10.1111/dom.13198 29272072

[31] Alvarez C Ramirez-Campillo R Martinez-Salazar C Mancilla R Flores-Opazo M Cano-Montoya J . Low-volume high-intensity interval training as a therapy for type 2 diabetes. Int J Sports Med. (2016) 37:723–9. doi: 10.1055/s-0042-104935 27259099

[32] Mangiamarchi P Caniuqueo A Ramírez-Campillo R Cárdenas P Morales S Cano-Montoya J . Effects of high-intensity interval training and nutritional education in patients with type 2diabetes. Rev Med Chil. (2017) 145:845–53. doi: 10.4067/s0034-98872017000700845 29182192

[33] Li R Li F . Effects of different exercise modes on blood glucose, blood lipid and other indexes in type 2 aged male diabetic patients. J Guangzhou Sport Univ. (2017) 37:99–101. doi: 10.3969/j.issn.1007-323X.2017.02.024

[34] de Oliveira VN Bessa A Jorge ML Oliveira RJ de Mello MT De Agostini GG . The effect of different trainin programs on antioxidant status, oxidative stress, and metabolic control in type 2 diabetes. Appl Physiol Nutr Metab. (2012) 37:334–44. doi: 10.1139/h2012-004 22458821

[35] Tan S Li W Wang J . Effects of six months of combined aerobic and resistancetraining for elderly patients with a long history of type 2 diabetes. J Sports Sci Med. (2012) 11:495–501. 24149359 PMC3737933

[36] Sigal RJ Kenny GP Boulé NG Wells GA Prud'homme D Fortier M . Effects of aerobic training, resistance training, or both on glycemic control in type 2 diabetes: a randomized trial. Ann Intern Med. (2007) 147:357–69. doi: 10.7326/0003-4819-147-6-200709180-00005 17876019

[37] Jorge ML de Oliveira VN Resende NM Paraiso LF Calixto A Diniz AL . The effects of aerobic, resistance, and combined exercise on metabolic control, inflammatory markers, adipocytokines, and muscle insulin signaling in patients with type 2 diabetes mellitus. Metabolism. (2011) 60:1244–52. doi: 10.1016/j.metabol.2011.01.006 21377179

[38] Wang C Zhang H . Influence of Baduanjin combined with routine treatment on blood glucose level in type 2 diabetic patients. China Med Pharm. (2015) 5:49–52.

[39] Liu H Chen Y Yi X Zhang Y Zhou Q Yu Y . Effect of Baduanjin exercise prescription on physical and mental regulation in type 2 diabetes patients with anxiety. Hunan J Traditional Chin Med. (2014) 30:16–8. doi: 10.16808/j.cnki.issn1003-7705.2014.07.007

[40] Duan J Li Z Li J . Clinical study of Baduanjin exercise prescription in the treatment of Type 2 diabetes. Chin Community Doctors. (2012) 14:218–9. doi: 10.3969/j.issn.1007-614x.2012.14.206

[41] Huang R Deng X . Treatment of type 2 diabetes with Baduanjin. Hebei J Traditional Chin Med. (2011) 33:1828–9. doi: 10.3969/j.issn.1002-2619.2011.12.037

[42] Li X . Effect of qigong·Baduanjin on endothelium-dependent arterial dilation of type 2 diabetes. J Shenyang Sport Univ. (2009) 28:50–51 + 55. doi: 10.3969/j.issn.1002-2619.2011.12.037

[43] Liu T Bai S Zhang R . Effects of Health Qigong Baduanjin on diabetes related indexes in middle-aged obese women. Chin J Appl Physiol. (2018) 34:19–22. doi: 10.12047/j.cjap.5484.2018.006 29926653

[44] Yavari A Najafipoor F Aliasgharzadeh A Niafar M Mobasseri M . Effect of aerobic exercise, resistance training or combined training on glycaemic control and cardiovascular risk factors in patients with type 2 diabetes. Biol Sport. (2012) 29:135–43. doi: 10.5604/20831862.990466

[45] Huang J . Effect of resistance-aerobic joint training at different proportion on blood glucose reduction of patients with type 2 diabetes mellitus. Modern Nurse. (2016) 03):8–10.

[46] Li T . Effects of different types of exercise on blood biochemical indexes of patients with type 2 diabetes. Shandong Sports Sci Technol. (2014) 36:81–4. doi: 10.3969/j.issn.1009-9840.2014.06.021

[47] Cheng H Shi J Weng Y LI W XU T . Influence of resistance training on glycometabolism and lipid metabolism in elderly patients with type 2 diabetes. J Clin Med Pract. (2013) 17:19–22. doi: 10.7619/jcmp.201323006

[48] Meng Q Chen W Zhang M Gao M . Effect of aerobic and resistance exercise for patients with type 2 diabetes mellitus. Chin J Rehabil Theory Pract. (2018) 24:1465–70. doi: 10.3969/j.issn.1006-9771.2018.12.021

[49] Chen SC Ueng KC Lee SH Sun KT Lee MC . Effect of t’ai chi exercise on biochemical profiles and oxidative stress indicators in obese patients with type 2 diabetes. JAltern Complement Med. (2010) 16:1153–9. doi: 10.1089/acm.2009.0560 20973735

[50] Lam P Dennis SM Diamond TH Zwar N . Improving glycaemic and BP control in type 2 diabetes. effectiveness tai chi Aust Fam Physician. (2008) 37:884–7. 19002314

[51] Meng En . Effect of Taichi on blood lipid composition and insulin resistance in patients with type 2 diabetes. Chin J Gerontol. (2014) 19):5358–60. doi: 10.3969/j.issn.1005-9202.2014.19.012

[52] Xiaobing Li . Effects of Taichi on oxidative stress and inflammation in elderly patients with type 2 diabetes. Chin J Gerontol. (2013) 33:5465–6. doi: 10.3969/j.issn.1005-9202.2013.21.132

[53] Nieuwoudt S Fealy CE Foucher JA Scelsi AR Malin SK Pagadala M . Functional high-intensity training improves pancreatic β-cell function in adults with type 2 diabetes. Am J Physiol Endocrinol Metab. (2017) 313:E314–320. doi: 10.1152/ajpendo.00407.2016 PMC5625086 28512155

[54] Wen J Lin T Cai Y Chen Q Chen Y Ren Y . Baduanjin exercise for type 2 diabetes mellitus: A systematic review and meta-analysis of randomized controlled trials. Evid Based Complement Alternat Med. (2017) 2017:8378219. doi: 10.1155/2017/8378219 29234435 PMC5671720

[55] Zhu S Wang C He J He . Meta-analysis of intervention effect of baduanjin on glucose and lipid metabolism in diabetic patients. Modernization Traditional Chin Med Materia Medica-World Sci Technol. (2020) 22:1478–86. doi: 10.11842/wst.20191108002

[56] Wang G Wang X Chen X . Meta-analysis of different forms of exercise effects on hbA1c percentage and part of body composition in patients with type 2 diabetes. China Sport Sci. (2016) 36:56–66. doi: 10.16469/j.css.201610008

[57] Wang Z Sun Pu Gguo C Tan X . Meta-analysis of the effect of traditional Chinese medicine on type 2 diabetes. Modern Prev Med. (2022) 49:1521–8.

[58] Tang Y Jin H Wu Y Fang C . Research progress of TaiChi as exercise prescription for type 2 diabetes mellitus. Lab Med Clinic. (2014) 2014:1105–6. doi: 10.3969/j.issn.1672-9455.2014.08.047

[59] Heiskanen MA Motiani KK Mari A Eskelinen JJ Virtanen KA Löyttyniemi E . Comment on ‘exercise training decreases pancreatic fat content and improves beta cell function regardless of baseline glucose tolerance: a randomised controlled trial’. Reply to Amini P and Moharamzadeh S [letter. Diabetologia. (2019) 62:204–6. doi: 10.1007/s00125-018-4762-4 30406809

[60] Liu X Miller YD Burton NW Brown WJ . A preliminary study of the effects of TaiChi and Qigong medical exercise on indicators of metabolic syndrome, glycaemic control, health-related quality of life, and psychological health in adults with elevated blood glucose. Br J Sports Med. (2010) 44:704–9. doi: 10.1136/bjsm.2008.051144 18927159

[61] Tan S Zhang Di Li W . Research on the effects of rehabilitation exercises on physical fitness of elder patients with type 2-diabetes. Chin J Rehabil Med. (2009) 24:719–22. doi: 10.3969/j.issn.1001-1242.2009.08.015

[62] Abd El-Kader SM Al-Jiffri OH . Impact of weight reduction on insulin resistance, adhesive molecules and adipokines dysregulation among obese type 2 diabetic patients. Afr Health Sci. (2018) 18:873–83. doi: 10.4314/ahs.v18i4.5 PMC6354881 30766550

[63] Zhang Y Cheng M . Adjustment effect of taijiquan on the old-aged’s blood greaseand adiposity diabete ii. J Beijing Sport Univ. (2008) 05):625–6. doi: 10.19582/j.cnki.11-3785/g8.2008.05.017

[64] Zhao G Chen M Zhuang Li Shun W . Effects of taijiquan on the physique, blood lipid, insulin resistance of patients with type 2 diabetes. J Nanjing Sports Institute. (2017) 16:1–7. doi: 10.15877/j.cnki.nsin.2017.01.001

[65] Guo H Li J Jiang Z . Follow-up effects of the increased physical activity on theglucolipid metabolic factors and medical costs in type 2 diabetic patients. Chin J Rehabil Med. (2007) 22:395–8. doi: 10.3969/j.issn.1001-1242.2007.05.009

[66] Guo Q Gan Y Zhang Y Zhou Y . Efficacy of resistance training on individuals with different glucose metabolism status: a meta-analysis. Chin J Evidence-Based Med. (2021) 21:1432–40. doi: 10.7507/1672-2531.202107086

